# Stress and serial adult metamorphosis: multiple roles for the stress axis in socially regulated sex change

**DOI:** 10.3389/fnins.2013.00210

**Published:** 2013-11-08

**Authors:** Tessa K. Solomon-Lane, Erica J. Crespi, Matthew S. Grober

**Affiliations:** ^1^Neuroscience Institute, Georgia State UniversityAtlanta, GA, USA; ^2^School of Biological Sciences and Center for Reproductive Biology, Washington State UniversityPullman, WA, USA; ^3^Department of Biology, Georgia State UniversityAtlanta, GA, USA

**Keywords:** hypothalamic-pituitary-adrenal/interrenal axis, sex change, corticotropin-releasing factor, cortisol, metamorphosis, stress

## Abstract

Socially regulated sex change in teleost fishes is a striking example of social status information regulating biological function in the service of reproductive success. The establishment of social dominance in sex changing species is translated into a cascade of changes in behavior, physiology, neuroendocrine function, and morphology that transforms a female into a male, or vice versa. The hypothalamic-pituitary-interrenal axis (HPI, homologous to HP-adrenal axis in mammals and birds) has been hypothesized to play a mechanistic role linking status to sex change. The HPA/I axis responds to environmental stressors by integrating relevant external and internal cues and coordinating biological responses including changes in behavior, energetics, physiology, and morphology (i.e., metamorphosis). Through actions of both corticotropin-releasing factor and glucocorticoids, the HPA/I axis has been implicated in processes central to sex change, including the regulation of agonistic behavior, social status, energetic investment, and life history transitions. In this paper, we review the hypothesized roles of the HPA/I axis in the regulation of sex change and how those hypotheses have been tested to date. We include original data on sex change in the bluebanded goby (*Lythyrpnus dalli*), a highly social fish capable of bidirectional sex change. We then propose a model for HPA/I involvement in sex change and discuss how these ideas might be tested in the future. Understanding the regulation of sex change has the potential to elucidate evolutionarily conserved mechanisms responsible for translating pertinent information about the environment into coordinated biological changes along multiple body axes.

## Introduction

The mechanisms underlying the initiation of teleost sex change remain of great scientific interest in part because this life history transition is often socially regulated. Following the removal of the dominant fish in the social group, the individual that establishes and maintains dominance over the group changes sex (Godwin, 2009). Across species, the social environment exerts a powerful influence over individual phenotype, behavior, neuroendocrine state, reproductive success, and survival (Wilson, 1980; Ellis, 1995; Adkins-Regan, 2009). Here, we highlight sex change as a unique opportunity to understand the dramatic and diverse biological processes regulated by the social environment.

In addition, sex change has important fitness consequences. Dominance in sex changing species offers a significant reproductive advantage, as it does in many non-sex changing species (Smuts and Smuts, 1993). Social groups often have a heavily skewed sex ratio, and those dominant individuals of the underrepresented sex reproduce with multiple subordinate group members. The ability to transition from a subordinate of one sex to the dominant of the other sex allows individuals to reproduce as female when young and small, for example, and as a male when older, larger, and able to defend a territory. Sex change thus results in an exponential increase in reproductive success (Ghiselin, 1969; Warner, 1975). Therefore, in understanding sex change, there is the potential to uncover mechanistic links connecting social information to the biological state of an individual to lifetime fitness.

In this paper, we review what is known about sex change in fishes, articulate the hypothesized roles for the hypothalamic-pituitary-adrenal axis (HP-interrenal in fish and amphibians; HPA/I) in sex change and how those hypotheses have been tested, and present original data that support roles for both cortisol and corticotropin-releasing factor (CRF) in both social hierarchy establishment/maintenance and sex change in the bluebanded goby (*Lythrypnus dalli*), a highly social, bidirectionally sex changing fish. We then synthesize our findings and propose future research directions that will more clearly elucidate the role(s) of the HPI axis in sex change.

## Sex change in teleost fishes

Teleost fishes display a remarkable amount of sexual plasticity, including the ability to sexually reorganize in adulthood (reviewed in Devlin and Nagahama, 2002; Godwin et al., 2003; Sadovy de Mitcheson and Liu, 2008; Kobayashi et al., 2013). Here, we focus on sex change in sequential hermaphrodites. Protogynous sex changing fish (e.g., wrasses, Labridae) can transition from a functional female (i.e., producing female gametes) to a functional male (i.e., producing male gametes). Protandrous species (e.g., clownfish, Pomacentridae) change from male to female, and bidirectionally sex changing species (e.g., gobies, Gobiidae) can change back and forth multiple times (i.e., serial adult metamorphosis). For each type of sequential hermaphrodite, individuals reproduce as male or female but not both simultaneously (see simultaneous hermaphrodite). Sex change involves coordinated biological changes along multiple body axes. Behavioral sex change is the earliest observable transition, often occurring within minutes in a permissive environment. During this phase, individuals adopt patterns of agonistic, courtship, and even reproductive behavior typical of the sex to which they are transitioning (Reavis and Grober, 1999; Godwin, 2009), changes that can occur independently of the gonad (Godwin et al., 1996). Physiologically, the most critical changes occur within the hypothalamic-pituitary-gonadal axis and result in the growth of new, and regression of old, gonadal tissue (Bass and Grober, 2001; Frisch, 2004; Godwin, 2009, 2010; Guiguen et al., 2010). Gonadal steroid hormones drive morphological sex change (Bass and Grober, 2001), which can range from relatively subtle changes in external genitalia for species that are not sexually dimorphic (St Mary, 1993) to dramatic changes in coloration and size (Godwin, 2009). Together, this coordinated cascade of behavioral, physiological, and morphological changes results in a functional female becoming a functional male, or vice versa.

Although previous research has elucidated many of the biological changes occurring during the different phases of sex change, particularly within the hypothalamic-pituitary-gondal axis (reviewed in Godwin, 2010), the biological signaling that interprets a permissive social environment and translates that information into the initiation of sex change has yet to be identified. A number of neuromodulators have been investigated as a potential biological switch important for the initiation of sex change, including neural steroid hormones (i.e., estradiol, 11-ketotestosterone, testosterone, cortisol) (Godwin, 2010; Lorenzi et al., 2012), gonadotropin-releasing hormone, arginine vasotocin (Reavis and Grober, 1999; Godwin et al., 2000; Bass and Grober, 2001), aromatase (Black et al., 2011), and serotonin (Lorenzi et al., 2009), with an increasing interest in kisspeptin (Godwin, 2010). Despite this focus, the biological link between social environment and sex change has not been resolved. Here, we address the roles that the HPA/I axis might play in the initiation or elaboration of adult sex change.

## Hypotheses for hypothalamic-pituitary-adrenal/interrenal axis regulation of sex change

The HPA/I axis has been implicated on multiple levels in the mechanistic control of sex change (e.g., Perry and Grober, 2003) because of its unique biological position translating environmental cues into biological responses (Lowry and Moore, 2006; Denver, 2009). In all vertebrates, the HPA/I axis integrates important internal and external information in response to environmental stressors, or external conditions that disrupt or threaten to disrupt homeostasis, and coordinates responses such as changes in behavior and physiology. In fish, CRF released from the hypothalamus signals the release of adrenocorticotropic hormone, which then initiates the release of glucocorticoids (GCs, e.g., cortisol) from the interrenal gland (Wendelaar Bonga, 1997; Mommsen et al., 1999). Previous research supports a role for the HPI axis in the regulation of three, non-mutually exclusive functions related to sex change: (1) social status, (2) agonistic behavior, and (3) life history transitions.

First, HPA/I axis activity plays a role in the establishment and maintenance of social status. In a range of social vertebrates, there are consistent differences in basal GC levels between dominant and subordinate social group members. Across species, dominants are almost equally likely as subordinates to have elevated GCs, and factors such as the distribution of resources, social stability, reproduction, and the nature of agonistic interactions among group members largely determine which status class is more socially “stressed” (Creel, 2001; Sapolsky, 2005). In cooperative breeders, for example, basal GCs are typically higher in dominant individuals (Creel, 2001). Correlations between social status and GCs have been reported in a number of teleosts, including rainbow trout (Øverli et al., 2004; Gilmour et al., 2005; Bernier et al., 2008), cichlids (Mileva et al., 2009), zebrafish (Filby et al., 2010), protandrous anemonefish (Iwata et al., 2012), and protogynous sandperch (Frisch et al., 2007). There are also status differences in brain CRF activity. In zebrafish, for example, CRF is more highly expressed in dominant hypothalamus but subordinate telencephalon (Filby et al., 2010). In rainbow trout, social subordination increases CRF expression in the preoptic area (Bernier et al., 2008), and in the cichlid *Astatotilapia burtoni*, transitioning from dominant to subordinate status results in a transient decrease in CRF, CRF receptor 2, and CRF binding protein (Chen and Fernald, 2011). Together, these data support HPI axis activity as an indicator of status that could be utilized in sex changing species to distinguish dominant from subordinate. A change in social status concurrent with a change in HPI function could play a role in the initiation of sex change.

Second, the HPA/I axis is implicated in the control of agonistic behavior. In mammals, fish, amphibians, and reptiles, neurons that express CRF are found throughout the brain in a conserved distribution (Lovejoy and Balment, 1999; Denver, 2009), and CRF signaling has highly conserved effects on arousal and anxiety-related behaviors, locomotion, exploration, and feeding (Koob and Heinrichs, 1999; Bale and Vale, 2004; Lowry and Moore, 2006). Exogenous manipulation of CRF signaling can also affect agonistic behavior and social status, although the direction of the effect is not fully resolved (Carpenter et al., 2009; Backström et al., 2011). The behavioral effects of CRF are likely mediated, in part, by monoamine signaling (e.g., serotonin, dopamine) (Summers and Winberg, 2006; Carpenter et al., 2009; Backström et al., 2011). At the level of GCs, fish that release greater amounts of cortisol in response to a stressor (high responsive) are consistently subordinate to low stress responders (Pottinger and Carrick, 2001), and individual variation in the amount of cortisol released in response to a stressor can be used to predict dominance outcome in a novel pair of fish (Øverli et al., 2004). Because agonistic behavior is critical during status establishment, and there are persistent behavioral differences among statuses in stable groups (Drews, 1993; e.g., Smuts and Smuts, 1993; Clarke and Faulkes, 2001; Graham and Herberholz, 2008), the role of the HPI/A axis in the control of agonistic behavior may be closely related to its role as a correlate of social status.

Third, the HPA/I axis could serve in an evolutionarily conserved role as a mediator of vertebrate life history transitions (Denver, 1999). Importantly, this role as a regulator of developmental plasticity seems to be independent of HPA/I-mediated responses to “unpredictable” environmental stressors, responses that might include the mobilization of energy reserves or altering behavior. Just prior to major transitions including birth/hatching (mammals, reptiles, birds, “large egg” fish), fledging (birds), dispersal (mammals, reptiles, birds), metamorphosis (amphibians), and smoltification (anadromous fish), both CRF and GCs have been shown to naturally increase (reviewed in Wada, 2008; Crespi et al., 2013). Exogenous elevation of GCs has also been used to initiate life history transitions (e.g., partuition in sheep), increase the success of the transition (e.g., hatching success in turkeys), and facilitate behavior (e.g., dispersal behavior in ground squirrels). In amphibians and fish, CRF stimulates the secretion of both thyroid hormone and GCs, which promote developmental transitions between life history stages and regulate developmental plasticity (e.g., amphibians: Denver, 1997; Boorse and Denver, 2004; Okada et al., 2007; fish: Larsen et al., 1998; Ebbesson et al., 2011) through both individual and synergistic actions (Hayes, 1997; Krain and Denver, 2004; Bonett et al., 2010; Kulkarni and Buchholz, 2012). Increases in thyroid hormone also precede reproductive maturation in fishes (also in mammals, Mann and Plant, 2010), and it has been hypothesized that serial adult sex change is simply the reoccurrence of these maturation processes (Dufour and Rousseau, 2007). Although there has been limited research on the thyroid axis and sex change (An et al., 2010; Park et al., 2010), sex change shares many characteristics of “classical” metamorphoses that are largely facilitated by actions of thyroid hormone and GCs. For example, smoltification in salmon, amphibian metamorphosis, and sex change all involve environmentally triggered morphological, physiological, and behavioral transformations in post-embryonic animals (Laudet, 2011). Therefore, we hypothesize that evolutionary conserved hormonal systems that mediate developmental plasticity, such as the HPA/I axis, are also acting during sex change in fishes.

It is important to note that for each hypothesized role for the HPI axis in sex change, HPA/I axis regulation of energy may also be relevant. Basal GCs are indicative of an individual's energetic demands and may be affected by time of day (e.g., appetite/foraging patterns), season (e.g., reproductive state), and life history stage. Stress-induced GC levels indicate the response of the HPA/I axis to an environmental challenge that requires energy mobilization to fuel behavioral and/or physiological responses (Sapolsky et al., 2000). In the case of social stressors, energetic demands may be elevated simply by the perception of dominant individuals (Sapolsky, 2005), and status differences in HPA/I activity could serve to facilitate differences in rates of behavior and/or reproductive demands. During life history transitions, changes in behavior, physiology, and morphology dramatically increase energetic requirements (Wada, 2008). For a transition like sex change, the exponential increase in reproductive success clearly outweighs energetic costs of sexual reorganization (Warner et al., 1975; Warner, 1984; Schreck, 2010). Finally, following a life history transition, HPA/I activity may be set to a new baseline because the energetic demands of the pre- and post-transition animal differ. In protogynous sex change, for example, reproductive investment might decrease because the energy required to produce sperm is traditionally considered lower than the energy to produce eggs. For some species, however, this difference may not be as sexually dimorphic as predicted (Yong and Grober, 2013), particularly for externally fertilizing species (Warner, 1997).

Together, these multiple lines of evidence strongly suggest a role for the HPI axis as a critical, proximate regulator of sex change. Here, we present original data that elucidates the roles of cortisol and CRF in a sex changing fish. All experiments were conducted in accordance with IACUC regulations and standards (Georgia State University, Atlanta, GA).

## Cortisol, social hierarchies, and sex change

Cortisol has been implicated in environmentally controlled sex determination in both gonochoristic (e.g., Hattori et al., 2009) and sex changing fish (Perry and Grober, 2003). For example, in gonochoristic fish with temperature-dependent sex determination, cortisol plays a critical mechanistic role in masculinization. At high water temperatures that normally cause testes to develop, pejerrey have elevated cortisol compared to fish at female-producing temperatures (Hattori et al., 2009). Exogenous cortisol administration can induce masculinization in the absence of high water temperatures in pejerrey (Hattori et al., 2009), Japanese flounder (Yamaguchi et al., 2010), and medaka, and an antagonist can prevent this masculinization (Hayashi et al., 2010). Cortisol seems to induce these changes through effects on enzymes involved in androgen pathways (Yamaguchi et al., 2010; Fernandino et al., 2012), and there is evidence in medaka that coritsol can also suppress feminization (Hayashi et al., 2010).

**Figure 1:**
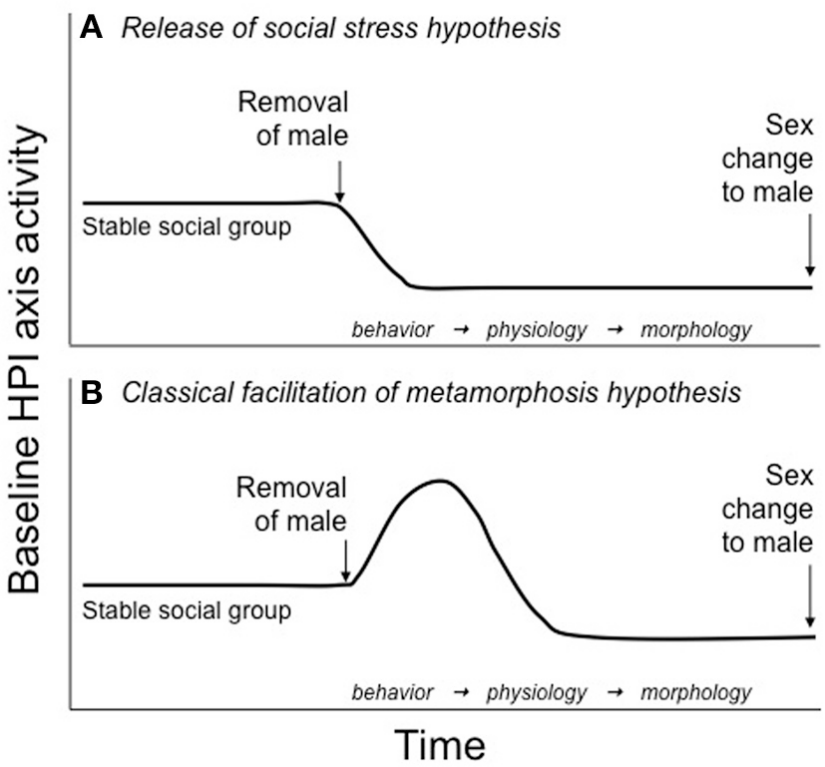
**Hypotheses for HPI axis involvement in teleost sex change. (A)** The release of social stress hypotheses, originally put forth by Perry and Grober (2003), suggests that baseline HPI axis activity is maintained at relatively constant, elevated levels in females of protogynous species because of the stress of subordination imposed by the dominant male. After male removal from the social group, HPI axis activity *decreases* in the most dominant female as she is released from the social stress of subordination. It is this decrease in HPI activity that allows for the initiation of sex change. Lower ranking females do not change sex because the dominant female/sex-changer maintains their subordinate status and, subsequently, their elevated baseline HPI axis activity. **(B)** The classical facilitation of metamorphosis hypothesis, discussed here for the first time, suggests that following male removal, baseline HPI axis activity *increases* in the dominant female/sex changer. Elevated HPI axis activity could be used to fuel the energetically costly changes in behavior, physiology, and morphology that occur during sex change and/or activate the thyroid axis, which could regulate cellular differentiation and apoptosis associated with sex change. This hypothesis is consistent with an evolutionarily conserved role for the HPA/I axis in the regulation of life history transitions.

In sex changing fish, cortisol could serve a similar role, linking environmental conditions to sexual differentiation. In one of the first mechanistic hypotheses, Perry and Grober (2003) suggested that dominant males of protogynous species prevent sex change in subordinate females via aggressive interactions that cause an increase cortisol levels. They hypothesized that this chronically elevated female cortisol is responsible for inhibiting sex change. If the dominant male were removed from the social group, then the most dominant female would be released from social subordination stress and her cortisol levels would decrease to male-typical levels. This release from social stress could trigger the initiation of sex change (Figure 1A). The remaining females in the social group would not change sex despite the permissive environment because aggression from the dominant female/sex changer would keep their cortisol elevated (Perry and Grober, 2003). Frisch et al. (2007) tested this hypothesis with the protogynous sandperch (*Parapercis cylindrica*) by inserting cortisol implants into the dominant female of a social group to prevent sex change once the male was removed. While the implants successfully elevated cortisol levels, they did not inhibit sex change (Frisch et al., 2007). If elevated CRF associated with social stress is involved in sex change, however, this manipulation would not recapitulate that condition. Interestingly, although designed to test the Release from Social Stress hypothesis (Figure 1A), the cortisol manipulation in Frisch et al. actually mimics the action of GCs in the competing hypothesis we present in this paper, the Classical Facilitation of Metamorphosis hypothesis (Figure 1B, discussed below). It cannot be determined from the data presented in Frisch et al. whether elevated cortisol facilitated or accelerated sex change, which could provide support for this alternative hypothesis.

We took a different approach to testing the Release from Social Stress hypothesis (Figure 1A) and measured endogenous cortisol levels in experimental social groups of another sex changing fish, the bluebanded goby (*L. dalli*). This small [standard length (SL) 18–45 mm] marine goby is highly social and lives on rocky reefs in the Pacific Ocean, from Morro Bay, California to as far south as the Galapagos Islands, Ecuador (Miller and Lea, 1976; Béarez et al., 2007). Mixed-sex social groups of *L. dalli* vary from small and isolated (3–10 fish) to aggregations of 120 fish/m^2^ (Steele, 1996) and are comprised of a dominant, territorial male and multiple subordinate females (St Mary, 1993). On the reef, *L. dalli* primarily undergoes protogynous sex change, and this could occur when a male is eliminated from his territory by predation or when multiple females converge on a territory without a male. In the laboratory, *L. dalli* is capable of both protogynous and protandrous sex change (e.g., bidirectional sex change, Rodgers et al., 2007).

**Figure 2:**
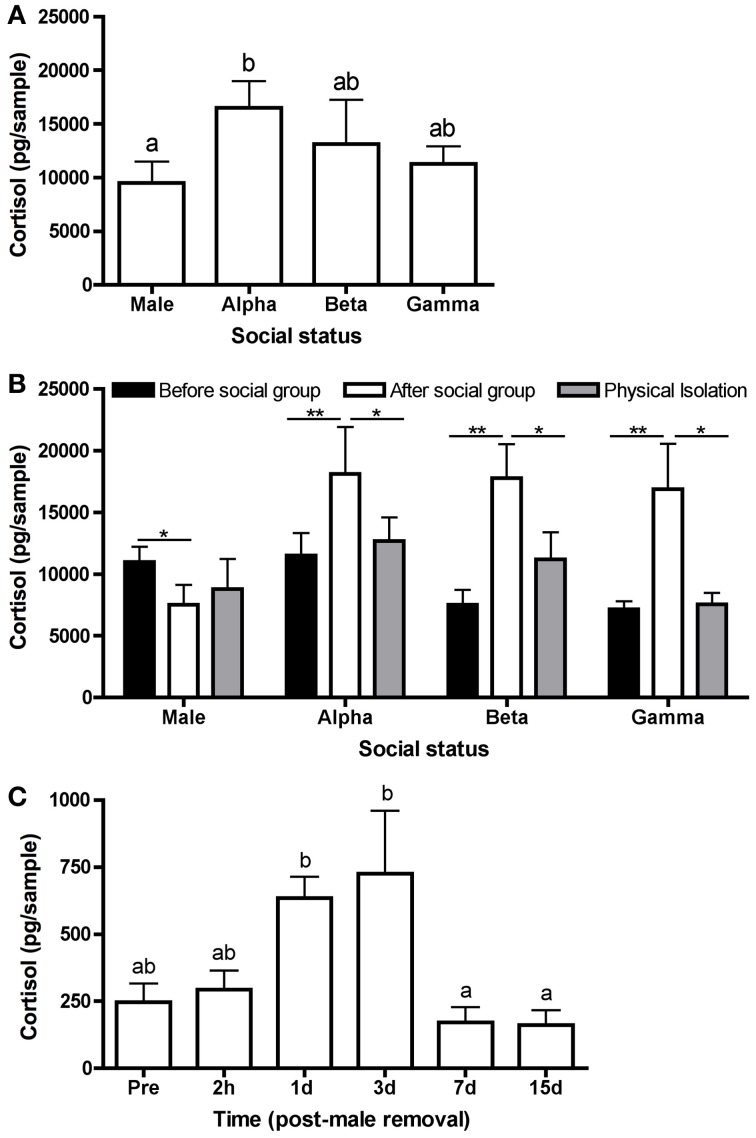
**Variation in water-borne cortisol levels. (A)** Mean (± s.e.m.) cortisol levels in each status class from stable social groups. Cortisol levels differed significantly among males, alpha females, beta females, and gamma females after 21 days in a stable social group (*n* = 39) (one-way ANOVA following a natural log transformation: *F*_(3, 149)_ = 3.64, *p* = 0.014). **(B)** Mean water-borne cortisol in males, alpha females, beta females, and gamma females prior to being placed in a social group (“before”) (*n* = 15), after 21 days in a social group (“after”) (*n* = 15), and following at least 1 day in a social isolation chamber (“physical isolation”) (*n* = 15). There were no differences in cortisol levels within statuses following 1, 2 or 3 days in the isolation chamber (*p* > 0.05); therefore, cortisol levels were pooled and presented in one column (“physical isolation”). Male cortisol “before” was significantly higher than “after” [paired *t*-test: *t*_(14)_ = 2.06, *p* = 0.05], whereas female cortisol was significantly higher “after” than “before” [paired *t*-tests: *t*_(14)_ > 2.8, *p* < 0.01]. Following isolation, cortisol levels were not significantly different from “before” for any social status [paired *t*-test: *t*_(14)_ < 1.8, *p* > 0.09]. For alpha, beta, and gamma females, “after” cortisol levels were significantly higher than isolation (paired *t*-tests: *t*_(14)_ > 1.9, *p* < 0.07); however, there was no difference for males [paired *t*-test: *t*_(14)_ = 0.52, *p* = 0.60]. **(C)** Mean water-borne cortisol differed significantly over time in alpha females in a stable social hierarchy (“pre”) (*n* = 12) and 2 h, 1, 3, 7, and 15 days (*n* = 12 each time point) following the removal of the male [one-way ANOVA: *F*_(5, 64)_ = 7.77,*p* < 0.0001]. Asterisks and different letters (*p* = 0.05) indicate significant differences.

The fish used in the following experiments were collected during the reproductive season from reefs offshore of Santa Catalina Island, CA using hand nets and SCUBA diving. For experiments described in Figures 2A,B, fish were then shipped to Georgia State University (Atlanta, GA) and housed communally before being placed into individual social groups (38 l aquaria). These tanks were maintained with artificial salt water and exposed to a 12:12 light-dark cycle. All other experiments took place at the Wrigley Institute for Environmental Studies (University of Southern California) on Catalina Island where water tables were continuously supplied with ocean water and exposed to a natural light cycle. To form social groups of specific sizes and sex ratios, fish were briefly anesthetized in tricaine methanesulfonate (MS-222), and we measured SL and determined sex based on genital papilla morphology (St Mary, 1993).

We conducted 3 experiments to determine whether elevated cortisol levels could be responsible for the chronic inhibition of sex change in female *L. dalli*. First, we tested whether cortisol levels were elevated in females compared with males by forming social groups (*n* = 39) of 1 large dominant male, 1 large dominant female (alpha; smaller and subordinate to the male), and 2 smaller females (beta and gamma). Hierarchies in these social groups were allowed to establish and be maintained for 21 days, and 10 min behavioral observations were conducted multiple times to verify hierarchy stability. On day 22, water-borne hormones were collected, a measure of systemic hormones closely related to hormone levels in circulation (i.e., plasma, Kidd et al., 2010). Fish of each social status were placed individually in a beaker of salt water for 1 h. Steroids were extracted from the water using C18 columns and measured using cortisol enzyme immunoassay kits (Cayman Chemical, Ann Arbor, Michigan) as in Lorenzi et al. (2008). The hormone pellet was resuspended in enzyme immunoassay buffer (5% EtOH), and we completed the assay according to the supplied instructions. All samples were assayed in duplicate.

We found that water-borne cortisol levels differed significantly among social statuses in stable *L. dalli* hierarchies. Cortisol levels were highest in the alpha females and lowest in males. Beta and gamma cortisol levels were intermediate between the males and alphas (Figure 2A). These results are consistent with the hypothesis of Perry and Grober (2003) that protogynous sex change could be inhibited in females via elevated cortisol, especially in the alpha female. Elevated alpha cortisol levels could result from aggression received from the male, the hybrid social position of being subordinate to the male yet needing to maintain dominance over the beta and gamma females, and/or increased energetic demands to fuel higher rates of agonistic interaction.

Next, we tested whether these status differences in cortisol were due to intrinsic differences rather than a consequence of the social hierarchy, as hypothesized. To determine whether social status drives differences in cortisol, we identified fish from the communal holding tank to form an additional 15 social groups and measured water-borne cortisol in those individuals prior to being placed in a social group, after 21 days in a social group, and then following 1 (*n* = 5), 2 (*n* = 5), or 3 (*n* = 5) days in isolation. The isolation chamber consisted of four compartments separated by glass partitions with 5 cm of space between each compartment. In this apparatus, the male and 3 females from a social group remained in visual and olfactory contact but could not physically interact. Fish were allowed to maintain some sensory contact because total isolation has been shown to independently increase cortisol in some individuals, particularly males (Earley and Grober, unpublished data). We found that before being placed into a social group, males had higher cortisol levels than after 21 days in a social group. Interestingly, females showed the opposite pattern and had higher cortisol levels after being in the social group. Following isolation, female cortisol decreased significantly to levels comparable to before being in the social group. Male cortisol levels did not change following isolation (Figure 2B). These data strongly suggest that the status- and sex-dependent cortisol differences after 21 days of interaction (Figure 2A) emerge as a consequence of the social environment rather than being reflective of intrinsic variation.

Finally, we tested whether cortisol decreases in the alpha female following the removal of the male. These data would provide support for the release of social stress initiating sex change (Figure 1A). To quantify cortisol over the course of sex change, we formed social groups (*n* = 60) of 1 large dominant male, 1 large dominant female, and 3 smaller females. Eight days after the groups were established, the male was removed to facilitate sex change in the alpha female. Water-borne cortisol was collected from the alpha female when the male was still present (*n* = 12) and then subsequently from alphas in different social groups (*n* = 12 each) 2 h, 1 day, 3 days, 7 days, and 15 days following male removal. Cortisol levels differed significantly over time, peaking 1–3 days after male removal (Figure 2C). These data show that contrary to the Perry and Grober (2003) hypothesis, cortisol in the alpha female/sex changer increased following the removal of the male. Interestingly, in the protandrous anemonefish (*Amphiprion melanopus*), cortisol levels do not differ between males in females but increases in the sex changer following the removal of the dominant female (Godwin and Thomas, 1993). This elevation in cortisol may be more consistent with the classical facilitation of metamorphosis hypothesis in which the HPA/I axis acts in a conserved role to facilitate vertebrate life history transitions (Figure 1B).

These data, in combination with Frisch et al. (2007), strongly indicate that there is no simple relationship between cortisol and sex change such that removing the male (the assumed source of elevated alpha cortisol) removes social subordination stress and leads to the initiation of sex change. Instead, cortisol may increase in the first few days of sex change, indicating increased CRF signaling and HPI axis activity. This increase could be necessary to meet the increased energetic demands involved with sex change and/or activate the thyroid axis, which could regulate cellular differentiation and apoptosis associated with sex change, similar to the gene programs activated by these axes during amphibian metamorphosis (Figure 1B). Interestingly, environmental cues such as increased density, reduced water volume, and reduced food availability activate the HPI axis in amphibian tadpoles and facilitate “stress-induced” metamorphosis (reviewed in Crespi and Denver, 2005). There could be a similar role for the HPI axis during sex change whereby a change in the environment (i.e., the removal of a social cue), or the behavioral changes induced by the environmental change, facilitates an important life history transition.

## Corticotropin-releasing factor, social status, and sex change

To further investigate the increase in HPI axis activity during sex change (Figure 2C), we focused on a role for CRF, the signal that drives the increase in cortisol. We hypothesized that a change in social environment, from a stable social group to an environment permissive for sex change, leads to an increase in CRF that could be involved in the establishment of dominance, agonistic behavior, and/or the metamorphic process of sex change (Figure 1B). To test these hypotheses, we exogenously elevated CRF using intracerebroventricular (icv) injection that was timed to coincide with a permissive environment: 2 size-matched female *L. dalli* in the absence of a male.

We collected females from reefs offshore of Catalina Island, CA and housed them in water tables at the Wrigley Institute for Environmental Studies. One day prior to pairing females, we briefly anesthetized fish (MS-222) to measure SL and mass. Paired females were size matched and differed in SL by an average of 0.19 ± 0.028 (s.e.m.) mm and differed in mass by 0.033 ± 0.0042 g. Females were then held in isolation overnight. The next morning, immediately prior to pairing, we used an established protocol for icv injection (Solomon-Lane and Grober, 2012) to acutely elevate CRF (Sigma-Aldrich, St. Louis, MO). Corticotropin-releasing factor-injected fish received 500 ng CRF/50.6 nL 0.1 M sterile phosphate buffer solution. All CRF-injected fish (*n* = 15) were paired with a size-matched female that received an injection of vehicle only (50.6 nL phosphate buffer) to control for the effects of injection during dominance establishment [similar design to Carpenter et al. (2009), Backström et al. (2011)]. Pairs of injected fish were compared to non-anesthetized, non-injected control pairs (*n* = 14). After both females in a pair recovered from the injection (described below), they were transferred simultaneously into a novel tank. Control females were not anesthetized and were transferred into the novel tank directly from their isolated housing.

Injections were performed using a Nanoject II Auto-Nanoliter Injector (Drummond Scientific Company, Broomall, PA, USA). Anesthetized fish were gently held under a dissecting microscope, and the pulled capillary tube needle attached to the Nanoject was lowered into position using a micromanipulator. The external anatomy of the head was used to correctly position the needle, and after penetrating the skull at the midline of the brain, the solution was injected into the third ventricle. Following injection, the needle was held in place for 5 s to reduce leakage, and after the needle was removed entirely from the fish, we performed a test injection to ensure that the needle was not clogged and to validate proper Nanoject function. Between injections, the needle was wiped with ethanol and allowed to dry, and the needle was changed between injections of CRF and vehicle. This technique has a success rate of at least 85% (Solomon-Lane and Grober, 2012) and has been used successfully in *L. dalli* to manipulate enzyme activity in the brain (Pradhan, Solomon-Lane, Willis, and Grober, in review).

**Figure 3:**
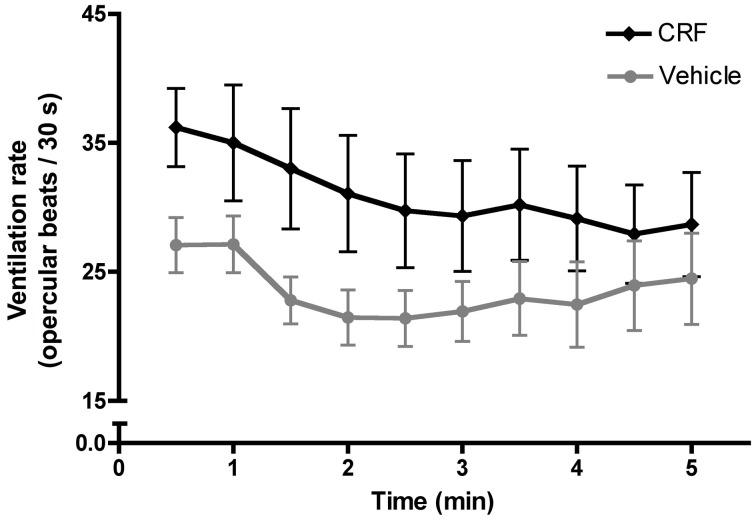
**Mean (± s.e.m.) ventilation rate, measured as the number of opercular beats recorded in 30 s intervals during the first 300 s following the initiation of ventilation.** A two-way mixed factorial ANOVA with time interval as the within subjects factor and treatment as the between subject factor demonstrated a significant effect of treatment [*F*_(1, 280)_ = 88.11,*p* < 0.0001] and time [*F*_(9, 280)_ = 3.187, *p* = 0.0011] on ventilation rate. *Post-hoc* analysis revealed that CRF-injected fish had a significantly higher ventilation rate in the first 300 s following the initiation of ventilation (independent *t*-test: *t* = 4.83, *d.f.* = 298,*p* < 0.0001), indicating the successful injection of CRF. *Post-hoc* testing showed no significant effect of time on ventilation rate (one-way ANOVA: *F*_(9, 290)_ = 0.78, *p* = 0.635).

Following injection, fish recovered in a 200 mL beaker of fresh salt water. Observing recovery provides independent verification that the injection procedure does not compromise an individual's locomotion or capacity for social interaction. Recovery from anesthesia is stereotyped and involves first initiating ventilation, indicated by movement of the opercula, and then regaining equilibrium, when the dorsal fin of the fish first reoriented to a vertical position. Observers were blind to the treatment of the recovering fish. Vehicle-injected fish did not differ from CRF-injected fish in the time required to initiate ventilation (Mann–Whitney *U*-test: *U* = 76.50, *n*_CRF_= 15, *n*_veh_= 15, *p* = 0.14) or regain equilibrium (independent *t*-test: *t* = 1.23, *d.f.* = 28, *p* = 0.23) following anesthetization and injection, suggesting that CRF did not negatively affect basal physiology or behavior. We also recorded ventilation rate for the first 300 s following the initiation of ventilation. This serves as a bioassay for injection efficacy because CRF has an evolutionarily conserved role in elevating ventilation rate. As previously shown in *L. dalli* (Solomon-Lane and Grober, 2012), CRF injection significantly increased ventilation rate compared to vehicle-injected fish (Figure 3), indicating the successful elevation of central CRF.

Injected females were paired as soon as both members of the pair had recovered fully (i.e., regained equilibrium) (CRF-injected: 16.2 ± 1.5 min post-injection; vehicle-injected: 22.8 ± 1.7 min post-injection). To pair females, we gently transferred each fish into a novel tank simultaneously. Tanks were supplied with a PVC tube (15.2 cm length, 1.9 cm diameter) that dominant *L. dalli* establish as their territory and males use as a nest. Following a 1 min acclimation period, we began behavioral observations (10 min each) and recorded approaches, when one fish swims directly toward any part of another fish's body, within 2 body lengths, and displacements, in which the approached fish retreats or swims away. We also recorded lateral displays, an escalated aggressive interaction. We conducted up to 3, rolling behavioral observations (maximum 30 min). If dominance was established and one fish displaced the other 5 times without being displaced itself, we did not conduct additional morning observations.

Overall, significantly fewer injected pairs than control pairs had an established dominant fish based on our original criteria (5 uninterrupted displacements) within the first 30 min of pairing (injected: 3 of 15 pairs; control: 12 of 14 pairs) (Chi-square: χ^2^ = 12.54, *d.f.* = 1, *p* < 0.001). Using a broader definition of dominance that included occupation of the nest territory and high agonistic efficiency, the proportion of approaches that lead to a displacement, a ratio that is substantially higher in dominants, there were still significantly fewer injected pairs with a clear dominant fish (injected: 7 of 15 pairs; control: 13 of 14 pairs) (Chi-square: χ^2^ = 7.24, *d.f.* = 1, *p* = 0.01). To determine if CRF facilitates dominance establishment in *L. dalli*, we compared the number of pairs with a CRF-injected and a vehicle-injected dominant fish. Of the 7 injected pairs with a clear dominant, only 1 dominant fish had been injected with CRF, which was not a significant difference from random (Chi-square: χ^2^ = 2.28, *d.f.* = 1, *p* = 0.13). During the afternoon observation, approximately 3 h after pairing, all 14 control pairs had an established dominant fish, defined by occupation of the nest territory and/or the consistent displacement of the subordinate fish. For the injected pairs, 13 of 15 pairs had an established dominant fish, and there was no difference between the number of CRF-injected (5) and vehicle-injected (8) dominants (Chi-square: χ^2^ = 0.3, *d.f.* = 1, *p* = 0.58).

These data demonstrate that contrary to our hypothesis, acute elevation of central CRF in this context did not facilitate dominance establishment. In fact, within the first 30 min of pairing, CRF-injected fish tended to become subordinate. In two similarly designed studies using juvenile rainbow trout (*Oncorhynchus mykiss*), CRF had conflicting effects on dominance establishment. Carpenter et al. (2009) showed that icv CRF at the same dose used in this study positively affected dominance establishment (Carpenter et al., 2009); however, Backström et al. (2011) report the same status outcomes after 60 min as we do for *L. dalli* during the afternoon observation: 5 CRF-injected and 8 vehicle-injected rainbow trout became dominant. Interestingly, at a higher dose, they show a negative effect of CRF on dominance (Backström et al., 2011). These data suggest that CRF may facilitate subordinate status, which could be confirmed for *L. dalli* with a larger sample size and/or by increasing the dose of CRF.

**Figure 4:**
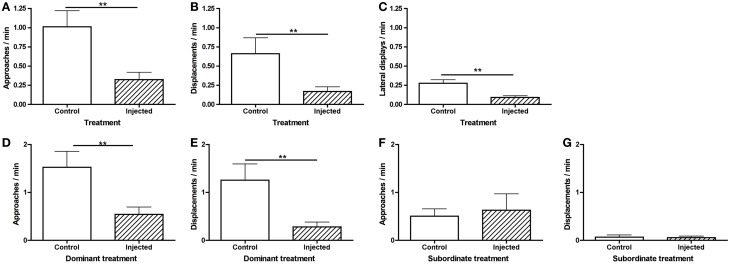
**Mean (± s.e.m.) agonistic behaviors within the first 30 min of pairing.** Injected fish **(A)** approached (independent *t*-test following natural log transformation: *t* = −3.09, *d.f.* = 44, *p* = 0.0035) and **(B)** displaced (Mann–Whitney *U*-test: *U* = 214.5, *n*_Inj_ = 30, *n*_Con_ = 26, *p* = 0.0039) significantly less than control fish. **(C)** Injected fish also engaged in significantly fewer lateral displays (independent *t*-test following natural log transformation: *t* = −3.53, *d.f.* = 24, *p* = 0.0017). Injected dominants **(D)** approached (independent *t*-test following natural log transformation: *t* = −3.22, *d.f.* = 18, *p* = 0.0079) and **(E)** displaced (Mann–Whitney *U*-test: *U* = 9.0, *n*_Inj_ = 7, *n*_Con_ = 13, *p* = 0.0043) significantly less than control dominants. There were no significant differences in subordinate **(F)** approaches (Mann–Whitney *U*-test: *U* = 38.5, *n*_Inj_ = 7, *n*_Con_ = 13, *p* = 0.61) or **(G)** displacements (Mann–Whitney *U*-test: *U* = 36.5, *n*_Inj_ = 7, *n*_Con_ = 13, *p* = 0.50). ^**^p < 0.01.

To investigate why status establishment was significantly delayed in injected pairs, we compared rates of agonistic behavior during the morning observations. Fish that were injected, including both CRF-injected and vehicle-injected fish, approached (Figure 4A) and displaced (Figure 4B) less than controls and engaged in fewer lateral displays (Figure 4C). These differences in agonistic behavior were driven specifically by dominants. Injected dominant fish (1 CRF, 6 vehicle) approached (Figure 4D) and displaced (Figure 4E) significantly less than control dominants, yet there were no differences in subordinate approaches (Figure 4F) or displacements (Figure 4G). These data demonstrate that the injection procedure, independent of substance injected, depressed behavior, which is critical to the establishment of dominance. Interestingly, this effect was mediated by social context: rates of behavior were depressed only in dominant fish.

**Figure 5:**
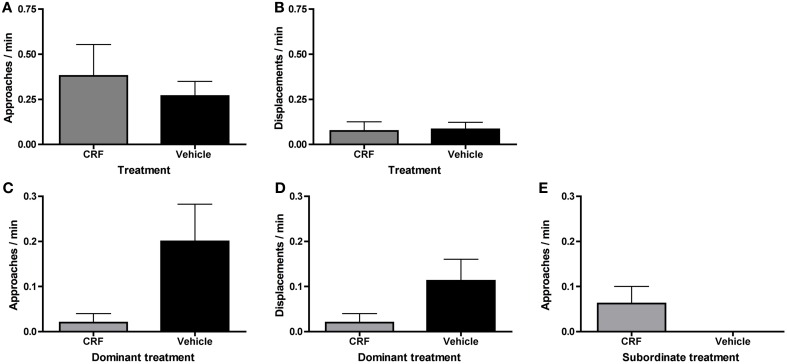
**Mean (± s.e.m.) agonistic behaviors in injected fish on day 1 of pairing.** There were no differences in **(A)** approaches (independent *t*-test following natural log transformation: *t* = 0.052, *d.f.* = 24, *p* = 0.95) or **(B)** displacements (Mann–Whitney *U*-test: *U* = 92.5, *n*_CRF_ = 15, *n*_Veh_ = 15, *p* = 0.41) by CRF-injected and vehicle-injected fish, independent of social status, within the first 30 min of pairing. In the afternoon observation, dominant CRF-injected fish did not **(C)** approach (Mann–Whitney *U*-test: *U* = 9.5, *n*_CRF_ = 5, *n*_Veh_ = 8, *p* = 0.13) or **(D)** displace (Mann–Whitney *U*-test: *U* = 12.5, *n*_CRF_ = 5, *n*_Veh_ = 8, *p* = 0.28) significantly more than vehicle-injected dominants. **(E)** There were not a sufficient number of subordinate approaches to analyze statistically, and subordinate displacements are not shown because all values are 0.

Although there was no significant difference in the number of CRF-injected vs. vehicle-injected dominants, vehicle-injected fish tended to become dominant in the first 30 min. To determine whether behavioral differences explain this skew and/or whether CRF affected agonistic behavior, we compared rates of approaches and displacements between CRF-injected and vehicle-injected fish, independent of status outcome. There were no differences in approaches (Figure 5A) or displacements (Figure 5B) in the first 30 min of pairing. Because there was only 1 CRF-injected dominant and only 1 vehicle-injected subordinate, we could not analyze whether agonistic behavior differed between CRF dominants and subordinates or vehicle dominants and subordinates. During the afternoon observation, when 5 CRF-injected and 8 vehicle-injected fish were dominant, there were no significant differences between CRF- and vehicle-injected dominants in approaches (Figure 5C) or displacements (Figure 5D). Although rates of behavior were lower in CRF-injected dominants, CRF did not seem to reduce agonistic efficiency: nearly all dominant approaches lead to a successful displacement. Among subordinates, there were no differences in approaches (Figure 5E) or displacements. Despite the low rates of subordinate behavior in the afternoon observation, CRF-injected subordinates interacted more than vehicle-injected subordinates, showing that CRF did not consistently decrease behavior further than the vehicle alone. Contrary to our hypothesis, therefore, exogenous elevation of central CRF did not affect agonistic behavior during status establishment and initiation of sex change. The lower rates of behavior due to injection, an effect also observed in rainbow trout injected with icv CRF (Carpenter et al., 2009), could also have limited our ability to detect differences.

**Figure 6:**
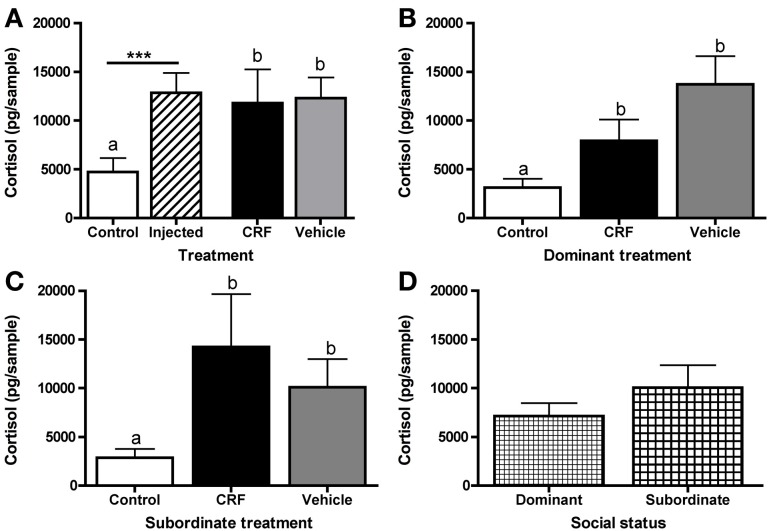
**Mean (± s.e.m.) water-borne cortisol levels (pg/sample). (A)** Injected fish had significantly higher cortisol levels than control fish (independent *t*-test following natural log transformation: *t* = 5.03, *d.f.* = 56>,*p* < 0.0001). Control, CRF-injected, and vehicle-injected fish cortisol also differed significantly (one-way ANOVA following natural log transformation: *F*_(2, 55)_ = 12.68,*p* < 0.0001). **(B)** Cortisol levels differed significantly among dominant (one-way ANOVA following natural log transformation: *F*_(2, 21)_ = 10.30, *p* = 0.0008) and **(C)** subordinate (one-way ANOVA following natural log transformation: *F*_(2, 21)_ = 8.98, *p* = 0.0015) fish. **(D)** There were no differences between all (control, CRF-injected, and vehicle-injected) dominant and all subordinate fish (independent *t*-test following natural log transformation: *t* = −0.18, *d.f.* = 46, *p* = 0.86). Asterisks and different letters (*p* < 0.05) indicate significant differences.

To test for an effect on HPI activity, resulting from the exogenous CRF and/or stress of the injection, on status establishment and agonistic behavior, we collected water-borne cortisol following the afternoon behavioral observation. Afterwards the pair was returned to their home tank. Injected fish had significantly higher cortisol levels than control fish, but there was no significant difference between CRF-injected and vehicle-injected fish (Figure 6A). This indicates that the injection rather than the exogenous CRF activated the HPI axis. For both dominants (Figure 6B) and subordinates (Figure 6C), CRF-injected and vehicle-injected fish had significantly higher cortisol than control fish but did not differ from each other. Despite similar HPI axis activation between injected groups, these data provide additional support for our ability to manipulate CRF centrally because the treatment difference in ventilation rate was not driven by a CRF effect on cortisol. Overall, there was no effect of social status on cortisol (Figure 6D).

Both the injection and the novel social environment, which was designed to be competitive by pairing size-matched females, likely contributed to the elevated cortisol levels, as both of these factors have been shown to activate the HPA/I axis. Interestingly, in a previous *L. dalli* study, CRF injected icv in the absence of a social manipulation did not affect cortisol levels when compared to anesthetized controls that were not injected (Solomon-Lane and Grober, 2012). This suggests a possible synergistic effect of the stressors. Elevated levels of CRF or cortisol also have been associated with suppression of behaviors, such as foraging, reproductive behaviors, and aggressive behaviors (Tokarz, 1987; Moore and Mason, 2001; Crespi and Denver, 2004). This is relevant to the present study because rates of behavior were depressed and cortisol levels were elevated in injected fish. Our inability here to distinguish between HPI activity in CRF-injected and vehicle-injected fish suggests that future tests of HPI involvement in *L. dalli* sex change may be limited by the current techniques. The challenge of manipulating the HPA/I axis without independently activating it via handling or administration procedure is frequently encountered by researchers, and we discuss potential future directions below.

**Figure 7:**
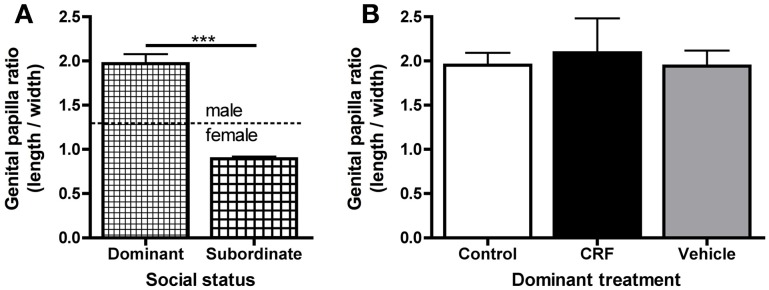
**Mean (± s.e.m.) genital papilla length-to-width ratio. (A)** Dominant fish (control, CRF-injected, and vehicle-injected) had significantly higher genital papilla ratios than subordinate fish (Mann–Whitney *U*-test: *U* = 12.0, *n*_dom_ = 25, *n*_sub_ = 27,*p* < 0.0001). Dominant fish had male-typical length-to-width ratios (average 1.99 ± 0.11) and subordinates had female-typical ratios (average 0.89 ± 0.023). **(B)** There were no differences in genital papilla ratio among dominant control, CRF-injected, or vehicle-injected fish [one-way ANOVA: *F*_(2, 22)_ = 0.099, *p* = 0.91]. Similarly, there was no difference among subordinates (data not shown, one-way ANOVA: *F*_(2, 24)_ = 0.22, *p* = 0.81). Asterisks indicate significant differences (*p* < 0.05).

Finally, to test whether CRF affected sex change, we allowed females to remain in their pairs for 12 days, a sufficient time for *L. dalli* to change sex (Reavis and Grober, 1999). By day 6, all control (*n* = 14) and injected (*n* = 15) pairs had a clear dominant fish, and except for status reversals in two control pairs, dominance remained stable through day 12. On day 6, CRF-injected fish had established and maintained dominance in 5 of 15 pairs, which was not significantly different from random (Chi-square: χ^2^ = 1.06, *d.f.* = 1, *p* = 0.30). We evaluated sex change from digital images of the sexually dimorphic genital papilla (St Mary, 1993) and gonad morphology. Female *L. dalli* have a rounded genital papilla with a length-to-width ratio less than 1.4, whereas the male papilla is pointed with a ratio greater than 1.4 (St Mary, 1993; Carlisle et al., 2000). In all pairs, the dominant fish changed sex, indicated by the significantly higher genital papilla ratio in dominant compared to subordinate fish (Figure 7A), which is consistent with many past studies in this species (Reavis and Grober, 1999; Rodgers et al., 2007). Visual inspection of the gonads also confirmed that all dominants had transitional or male-typical gonads. Among dominants, there was no effect of treatment on the genital papilla ratio (Figure 7B) suggesting that neither a single icv injection of CRF nor the injection procedure, both of which delayed status establishment and elevated cortisol, affected the rate of sex change.

## Summary and future research directions

In this paper, we have discussed multiple hypotheses about HPI axis involvement in the regulation of teleost sex change and presented original data from our initial experiments with *L. dalli* that test those hypotheses. We have shown that in *L. dalli*, variation in HPI axis activity (measured as cortisol) was associated with social status in the hierarchy, and these differences were socially mediated. We also showed that cortisol increased during early stages of sex change, suggesting that activation of the HPI axis may be involved with stimulating the process of sex change similar to the ways in which HPA/I axis activation is involved in other life history transitions (Wada, 2008). Additional experiments suggested below could elucidate whether the HPI axis is acting in this evolutionarily conserved manner during sex change.

Corticotropin-releasing factor, either through its hypophysiotopic actions or its own actions as a neurotransmitter, also could facilitate sex change through two possible scenarios. First, naturally occurring sex change is always coupled with social dominance; therefore, if CRF facilitated dominance, CRF could indirectly mediate sex change. We showed that this is not the case for *L. dalli*. Acute elevation of CRF in the brains (through icv injection) of fish in a permissive environment was not associated with dominance establishment or the expression of agonistic behavior, although there may be a role for CRF (and cortisol) in subordinate status and/or behavior. Instead, both icv CRF and vehicle reduced agonistic behaviors and delayed dominance establishment. Acute increases in CRF resulting from environmental stressors (e.g., predation threat, threatening abiotic conditions) could inhibit aggression because this switch in behavior favors survival in such conditions, as it does in other vertebrates (e.g., Tokarz, 1987), independent of its role in the regulation of behaviors that maintain hierarchies. More research is needed to examine both of these hypotheses.

Second, if CRF was the biological signal for the initiation of sex change, then CRF could directly regulate this process, possibly even in the absence of social dominance. It is important to note that since sex change is socially regulated by nuanced interactions among several individuals, there may not be one agonist or antagonist, within or outside of the HPI axis, capable of overriding the effects of social interactions and context. While we showed that a single icv injection of CRF prior to status establishment in a permissive environment did not trigger sex change, this does not necessarily rule out a role for CRF in its initiation. For example, it is likely that a prolonged elevation of hypothalamic CRF is necessary to activate the physiological hormonal cascade involved in sex change, as we detected elevated cortisol in alpha females days after removal of a dominant male. Indeed, Denver (1997) used repeated intraperitoneal (ip) injections of CRF to initiate precocious metamorphosis in tadpoles beyond a certain stage of development, and conversely, used repeated ip injections of a CRF antagonist to prevent metamorphosis.

Similar experiments could be conducted in *L. dalli* to test whether CRF administered to a dominant female in a permissive environment for sex change can accelerate the transition. This could be accomplished using multiple ip injections, which would allow for CRF to have hypophysiotropic effects (Denver, 1997) but may be less stressful than icv injections because less handling is required and anesthesia may not be necessary. Alternatively, icv injection of CRF in a viral vector could activate CRF over a longer period of time, thereby allowing fish to more fully recovery from the injection procedure before being exposed to a social challenge or an environment permissive to sex change. Repeated icv injections via indwelling cannula would also be possible in larger species of sex changing fish (e.g., wrasses, Labridae; parrotfishes, Scaridae). Another critical experiment would involve chronically inhibiting CRF in an environment permissive to sex change via administration of an antagonist (e.g., alpha-helical CRF), *vivo* morpholino (e.g., Ferguson et al., 2013), siRNA, or shRNA.

Overall, we encourage further investigation into the mechanisms underlying sex change in order to broadly elucidate social regulation of metamorphic processes, and, more specifically, identify a potentially evolutionarily conserved role for the HPI axis in this dramatic life history transition.

### Conflict of interest statement

The authors declare that the research was conducted in the absence of any commercial or financial relationships that could be construed as a potential conflict of interest.
